# Levosimendan *vs.* Intra-Aortic Balloon Pump in Coronary Artery Bypass Grafting: A Meta-Analysis

**DOI:** 10.21470/1678-9741-2025-0057

**Published:** 2026-02-13

**Authors:** Yanjie Wang, Jinluan Qu, Dan Sheng, Xiang Sun, Liqin Zhong, Yingjie Wu, Hao Liang

**Affiliations:** 1 Institute of Traditional Chinese Medicine Diagnostics, Hunan University of Chinese Medicine, Changsha, Hunan, People’s Republic of China; 2 School of Integrated Chinese and Western Medicine, Hunan University of Chinese Medicine, Changsha, Hunan, People’s Republic of China; 3 Cardiology Department, Changsha Hospital of Traditional Chinese Medicine, Changsha, Hunan, People’s Republic of China

**Keywords:** Simendan, Atrial Fibrilation, Arterial Pressure, Mediastinitis, Hospital Mortality, Coronary Artery Bypass, Cardiovascular Agents.

## Abstract

**Objective:**

To compare the clinical efficacy and safety of intra-aortic balloon pump (IABP) and levosimendan in coronary artery bypass grafting (CABG).

**Methods:**

A systematic search of PubMed®, Embase, Cochrane Library, and Google Scholar was conducted through July 2024. Outcomes analyzed included atrial fibrillation, postoperative mediastinitis, the requirement for inotropic support, in-hospital mortality, postoperative intensive care unit (ICU) stay, postoperative length of stay, ventilation time, and mean arterial pressure (MAP) levels.

**Results:**

The analysis included nine studies with 681 patients. Levosimendan presented advantage over IABP in CABG patients in terms of postoperative ICU stay, postoperative length of stay, and reduction in MAP levels, with effect sizes: mean difference (MD) = -0.83, 95% confidence interval (CI) -0.97 to -0.68, P < 0.00001, MD = -1.14, 95% CI: -1.33 to -0.95, P < 0.00001, and MD = -4.55, 95% CI: -6.14 to -2.96, P < 0.00001, respectively. Levosimendan had an advantage on subgroup analyses in terms of postoperative ICU stay and postoperative length of stay, with effect sizes: MD = -0.83, 95% CI: -0.93 to -0.72, P < 0.00001 and MD = -1.14, 95% CI: -1.28 to -1.01, P < 0.00001, respectively. However, the incidence of postoperative mediastinitis was higher in the levosimendan group (relative risk = 1.45, 95% CI: 0.88 to 2.38), though not statistically significant.

**Conclusion:**

Levosimendan may improve recovery and hemodynamic outcomes in high-risk CABG patients compared to IABP but may be associated with a higher, though non-significant, risk of mediastinitis. Further high-quality studies are warranted.

## INTRODUCTION

**Table:** 

Abbreviations, Acronyms & Symbols				
AF	= Atrial fibrillation		LVEF	= Left ventricular ejection fraction
BMI	= Body mass index		MAP	= Mean arterial pressure
CABG	= Coronary artery bypass grafting		MD	= Mean difference
CAD	= Coronary artery disease		MI	= Myocardial infarction
CI	= Confidence interval		NRCTs	= Non-randomized controlled trials
CO IABP	= Cardiac output = Intra-aortic balloon pump		PRISMA	= Preferred Reporting Items for Systematic Reviews and Meta-Analyses
ICU	= Intensive care unit		RCT	= Randomized controlled trial
IV	= Intravenous injection		RR	= Relative risk
LAD	= Left anterior descending artery		SD	= Standard deviation
LIMA	= Left internal mammary artery		SE	= Standard error

Coronary artery bypass grafting (CABG) is an effective surgical therapy for treating coronary artery disease (CAD), especially in patients with complex lesions and high-risk conditions^[1,2]^. By reconstructing hemodynamics, CABG improves blood circulation, reduces the risk of myocardial infarction (MI), and enhances myocardial blood supply by redirecting blood flow through alternative arteries^[3]^. Recanalization of the distal end of a blocked vessel following CABG can alleviate myocardial ischemia and hypoxia^[4]^. This procedure involves using arteries or veins from other parts of the patient's body to serve as a "bridge" connecting the proximal and distal ends of a narrowed coronary artery segment, effectively bypassing the obstruction to improve blood supply to the ischemic myocardium. However, post-CABG cardiac rehabilitation is a complex process, and optimizing cardiac recovery while minimizing complications remains a significant consideration.

Levosimendan is a novel positive inotropic medication that differs from other inotropic agents by enhancing cardiac myocytes' sensitivity to calcium through binding to troponin C. Additionally, it increases their sensitivity to calcium ions^[5]^. The administration of levosimendan during cardiac surgery can protect the heart, reduce ischemic or reperfusion injury, improve postoperative cardiac function, and effectively prevent and treat postoperative cardiac complications.

Intra-aortic balloon pump (IABP) stands as the most accessible circulatory support modality for patients with a history of cardiac surgery^[6]^. Its preoperative deployment in high-risk individuals positions IABP as a potential frontline therapy, correlating with enhanced survival outcomes^[7,8]^. As the most commonly employed mechanical circulatory assist device, IABP operates by inflating during diastole to augment cerebral, coronary, and systemic perfusion, while deflating during systole to reduce afterload, thereby boosting cardiac output (CO) and improving overall circulation.

The prognosis of CABG patients has been reported to be improved by the prophylactic use of IABP. But the main disadvantage of IABP is the emergence of problems related to balloon fitting, such as limb ischaemia and vascular damage, particularly in patients with systemic atherosclerosis. Levosimendan, a brand-new drug for the treatment of high-risk cardiac patients, has been shown to improve intraoperative hemodynamic parameters and reduce the incidence of postoperative low CO syndrome in many earlier studies, which in turn lowers the incidence of postoperative mortality, improves prognosis, and has fewer side effects. We therefore did a meta-analysis to assess the clinical efficacy and safety of levosimendan and IABP with the goal of supplying an evidence-based basis for clinical drug selection in order to identify the difference in complication rates between these two regimens.

## METHODS

In accordance with the Preferred Reporting Items for Systematic Reviews and Meta-Analyses (PRISMA) recommendations, we carried out a systematic review and meta-analysis of randomized trials. The registration number for the current study in PROSPERO is CRD42023444015.

### Search Strategy

Two authors (Yj. W. and Jl. Q.) conducted a comprehensive search of PubMed®, Embase, Cochrane Database of Clinical Trials, and Google Scholar for clinical trials on the use of levosimendan and IABP support in adult patients having CABG from database inception to July 2024. The Medical Subject Headings terms "coronary artery bypass grafting" ("CABG"), "levosimendan", "simendan", "intra-aortic balloon pump", and "IABP" were systematically searched in these databases. The specific search strategy is shown in Supplementary File 1. This meta-analysis had no language constraints, thus any publications written in other languages but with English abstracts were included, and their English versions were translated for further screening.

### Study Selection

Two authors (Yj. W. and Jl. Q.) used a questionnaire to initially screen all pertinent papers for titles and abstracts. Discussion and agreement were used to settle any differences. The authors of any papers that were eligible were then contacted via email to request the full text if it wasn't already online before the articles were included in a full-text review. Adult patients (> 18 years) undergoing CABG (on-pump or off-pump) and perioperative administration of levosimendan or an IABP with no restrictions on the dose or time of administration were the inclusion criteria. Atrial fibrillation (AF), postoperative mediastinitis, the requirement for inotropic support, in-hospital mortality, postoperative intensive care unit (ICU) stay, postoperative length of stay, ventilation time, and mean arterial pressure (MAP) levels were the main outcomes in this meta-analysis.

### Data Extraction

To ensure the accuracy and completeness of the data, the following key information was extracted from each study: study’s authors, year of publication, sample size, patient’s age, body mass index, left ventricular ejection fraction (LVEF), and prevalence of hypertension and diabetes. These data provide basic characteristics about the study population that are essential for subsequent analyses and comparisons. Specific data are detailed in Table 1.

**Table 1:** Basic information of the included studies.

Author	Year	N		Age (years)		BMI (kg/m^2^)		LVEF (%)		Hypertension (%)		Diabetes mellitus	
		Levosimendan	IABP	Levosimedan	IABP	Levosimendan	IABP	Levosimendan	IABP	Levosimendan	IABP	Levosimendan	IABP
Lomivorotov ^[9]^	2012	30 (24/6)	30 (29/1)	57.3 ± 8.6	56.8 ± 9.4	27.8 ± 5.4	28.8 ± 4.0	31 (28-33)	30 (29 - 33)	17 (57)	20 (66)	2 (30)	4 (30)
Alaa Omar ^[10]^	2020	135 (98/37)	144 (120/24)	57.7 ± 4.8	58.8 ± 4.2	30 ± 3.4	29 ± 3.5	32.2 ± 2.1	33 ± 1.7	81 (60)	101 (70)	76 (135)	91 (14 4)
Ritesh Mate ^[11]^	2020	30 (23/7)	30 (21/9)	60.2 ± 5.7	161.2 ± 8.5	65.6 ± 6.3	66.6 ± 6.9	20.5 ± 4.4	20.4 ± 4.52	19 (30)	20 (30)	21 (30)	22 (30)
Azzab ^[14]^	2021	30 (24/6)	30 (27/3)	57.7 ± 4.8	58.8 ± 4.2	30.0 ± 3.4	29.0 ± 3.5	4.32 ± 2.1	33.1 ± 1.7	18	21	21	23
Hady GA ^[13]^	2021	30 (20 /10)	30 (21 /9)	61.9 ± 13.4	53.3 ± 10.3	29.0 ± 3.3	28.3 ± 3.0	33	30	16 (51)	14 (49)	15 (50)	18 (60)
Allama ^[16]^	2020	30 (24/6)	30 (22/8)	58.3 ± 4.4	57.7 ± 4.8	30.3 ± 2.5	29.4 ± 3.4	32.1 ± 2.3	32.7 ± 2.1	20	18	17	17
Ragheb ^[12]^	2023	25	25	61.2 ± 8.5	60.2 ± 5.7	N/A	N/A	29.9 ± 4.5	30.3 ± 3.4	N/A	N/A	N/A	N/A
Severi ^[15]^	2011	11 (10/1)	11 (10/1)	60.0 ± 6.0	66.0 ± 12.6	N/A	N/A	26 ± 6.2	30 ± 6.4	6	5	5	4
Fawzy ^[17]^	2013	15 (9/6)	15 (11/4)	60.1 ± 7.6	59.3 ± 6.6	N/A	N/A	31.4 + 2.6	30.9 + 2.7	N/A	N/A	N/A	N/A

BMI=body mass index; IABP=intra-aortic balloon pump; LVEF=left ventricular ejection fraction; N/A=not applicable

### Statistical Analysis

A software called RevMan 5.4 was used to evaluate each observation. A fixed effects model was applied, and Cochrane Q was used to examine the heterogeneity of the included literature. *P* ≥ 0.10 and *I^2^* ≤ 50% demonstrated low heterogeneity between studies. If *I^2^* > 50%, the cause of the heterogeneity was investigated first; if the investigation was unsuccessful, a descriptive analysis was utilized instead of a meta-analysis. Variables in this meta-analysis included both continuous and dichotomous ones. Continuous variables were statistically analyzed using the standardized mean squared deviation, and all statistical analyses were expressed with 95% confidence intervals (CI). A funnel plot was used to display bias in publications.

## RESULTS

**Fig. 1:**
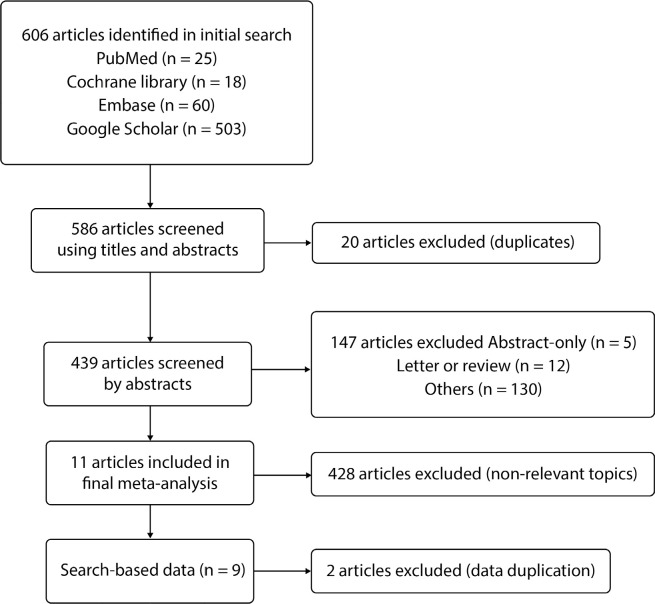
Flow diagram of studies selection.

The four abovementioned databases yielded a total of 543 records. Nine publications were included after screening of title, abstract, and full-text. Figure 1 displays the PRISMA flowchart for this investigation.

### Characteristics of Included Studies

With a total sample size of 681 cases from eleven studies^[9-17]^, 345 from the levosimendan group and 336 from the IABP group, this meta-analysis covers nine trials. Separate data (author, year, sample size of the research and control groups, patient’s age, etc.) was taken from each of the included articles. The data was retrieved, and Figure 1 shows that the patients with reduced left ventricular function had relatively old mean ages.

### Quality Assessment

**Fig. 2:**
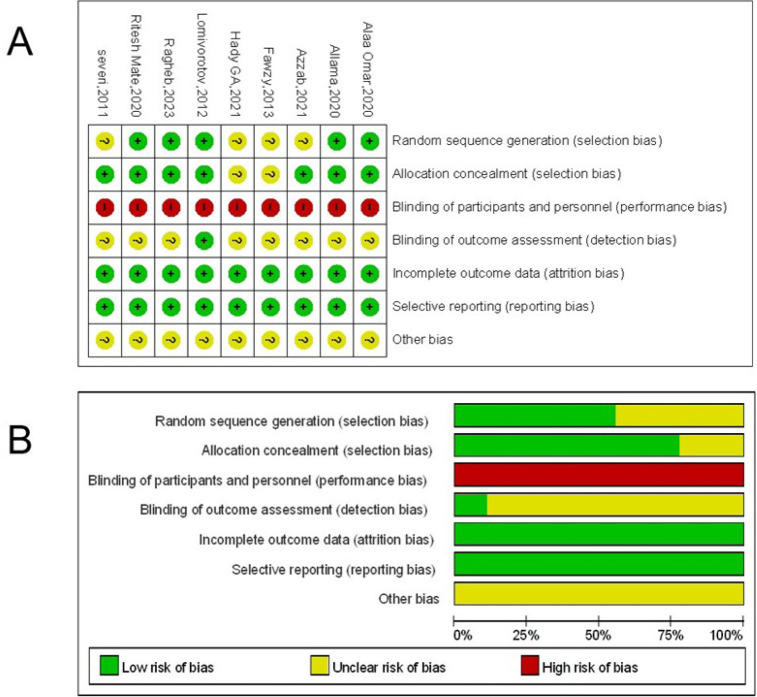
Risk of bias summary.

Two reviewers (D. S. and Yj. W.) independently evaluated the internal validity and risk of bias using defined criteria in accordance with Cochrane Methods. Six criteria were evaluated: sufficient sequence creation, concealment of allocation, blinding, insufficient outcome data, selective reporting, and absence of other biases. We stipulated that good-quality studies have a high or uncertain risk of bias in no more than two areas, as shown in Figure 2.

Several of the included studies demonstrated high or unclear risk of bias in critical domains, particularly in allocation concealment and blinding of participants and personnel. These issues were more prominent in non-randomized controlled trials (NRCTs), where lack of proper allocation procedures and performance bias could not be excluded. Although we attempted to assess all trials using the Cochrane Risk of Bias tool, the presence of such methodological limitations introduces a potential source of systematic error that may distort treatment effect estimates.

### Meta-Analysis

#### Atrial Fibrillation

**Fig. 3:**
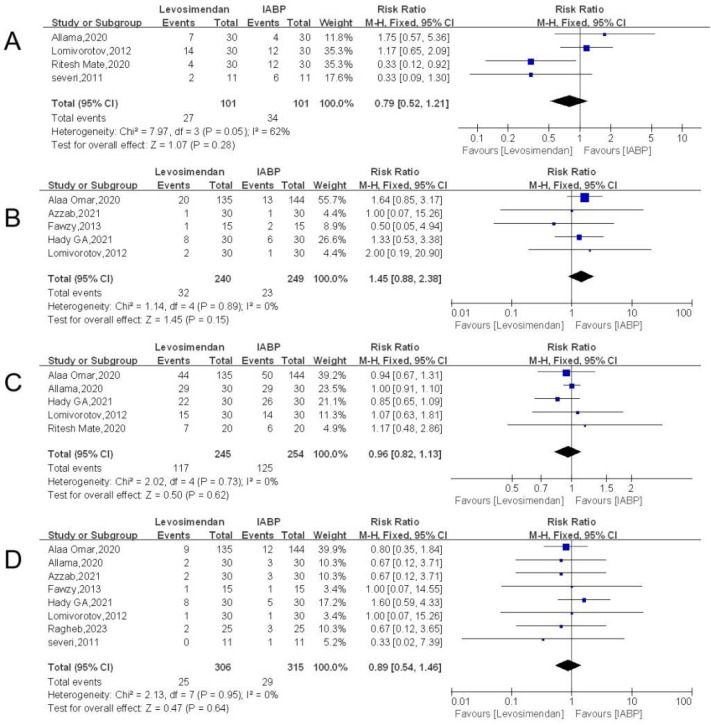
A) Forest plot of the incidence of atrial fibrillation. B) Forest plot of the incidence of mediastinitis. C) Forest plot of the need for inotropic support. D) Forest plot of in-hospital mortality. CI=confidence interval; IABP=intra-aortic balloon pump.

In four of the included publications, a statistical analysis of 202 people who had diminished left ventricular function used the incidence of AF as an outcome indicator. The levosimendan group had 101 cases, whereas the IABP group had 101 cases. In a meta-analysis utilizing relative risk (RR) as the effect size and the Q-test for heterogeneity, inconsistent results across effect sizes were discovered (*I^2^* = 62%), indicating significant study heterogeneity. So, a fixed effects model was used in the analysis. The results of the meta-analysis were as follows: effect value (RR = 0.79, 95% CI: 0. 52 to 1. 21, *P* = 0.89) (Figure 3A) with the diamond in the center of the chart, indicating no statistically significant difference between the two groups (*P* > 0.05).

### Postoperative Mediastinitis

In five of the articles, 489 patients with impaired left ventricular function were statistically analyzed using postoperative mediastinitis as an outcome indicator. The levosimendan and IABP groups had 240 and 249 of these patients, respectively. The analysis used a fixed effects model according to no heterogeneity (*I^2^* = 0%), and the findings of the meta-analysis were as follows: the diamond is situated on the right side of the axis, with an effect size of RR = 1.45, 95% CI: 0.88 to 2.38, *P* = 0.15 (Figure 3B). There was a statistically significant difference between the two groups (*P* > 0.05), the fact that postoperative mediastinitis was more frequent in the levosimendan group than in the IABP group.

### Need for Inotropic Support

The necessity for inotropic assistance was included as an outcome indicator in five of the included papers' statistical analyses of 499 patients with reduced left ventricular function. Of these, the levosimendan and IABP groups had 245 and 254 instances, respectively. The studies exhibited no significant heterogeneity (*I^2^* = 0%). A fixed effects model was used for analysis, and the results of the meta-analysis with RR as the effect size were as follows: effect size RR = 0.96, 95% CI: 0.82 to 1.13, *P* = 0.62 (Figure 3C), with the diamond intersecting the axis and located to the right of the axis. Overall, there was no significant difference between levosimendan and IABP in terms of need for inotropic support (*P* > 0.05).

### In-Hospital Mortality

Eight of the included articles statistically analyzed 709 patients with impaired left ventricular function, using in-hospital mortality as an outcome indicator. Of these, 350 and 359 cases were in the levosimendan and IABP groups, respectively. Meta-analysis with RR as the effect size and a Q-test for heterogeneity revealed heterogeneous results between effect sizes (*I^2^* = 0%), indicating no heterogeneity between studies, so a fixed effects model was used for the analysis, and the results of the meta-analysis were as follows: effect size RR= 0.89, 95% CI: 0.54 to 1.46, *P* = 0.64 (Figure 3D), with a diamond intersecting the axis. In-hospital mortality did not show a significant difference between the levosimendan group and the IABP group, and there was no statistically significant difference between the two groups (*P* > 0.05).

### Postoperative Intensive Care Unit Stay (days)

**Fig. 4:**
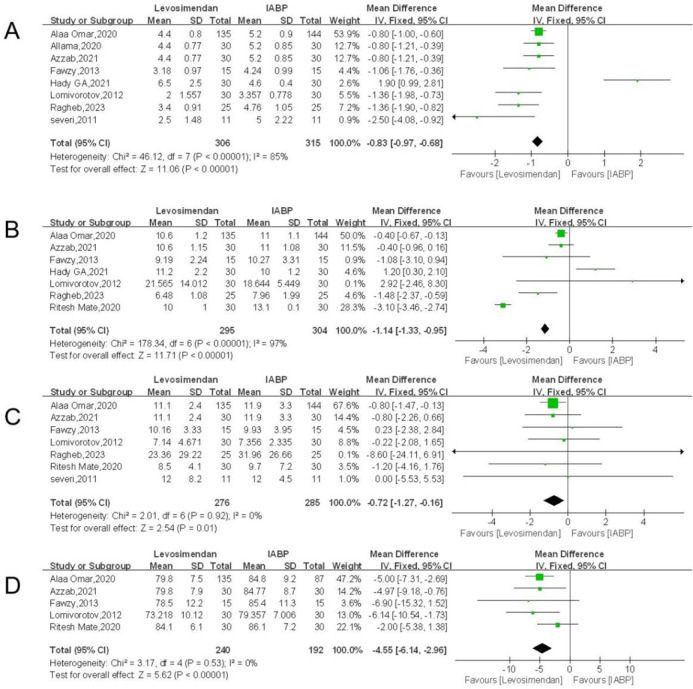
A) Forest plot of postoperative intensive care unit stay. B) Forest plot of postoperative length of stay. C) Forest plot of ventilation time. D) Forest plot of mean arterial pressure levels. CI=confidence interval; IABP=intra-aortic balloon pump; IV=intravenous injection; SD=standard deviation.

The postoperative ICU stay was used as an outcome indicator in eight of the included articles' statistical analyses of 621 patients with decreased left ventricular function. In the levosimendan and IABP groups there were 306 and 315 instances, respectively. Meta-analysis was conducted using mean difference (MD) as the effect size measure, and heterogeneity was evaluated. A random-effects model was chosen for the analysis because the Q-test revealed significant inter-study variability across effect sizes (*I^2^* = 85%). The meta-analysis' findings were as follows: the duration of postoperative ICU stay was shorter in the levosimendan group than in the IABP group, with a statistically significant difference between the two groups, effect size MD = - 0.83, 95% CI: -0.97 to -0.68, *P* < 0. 00001 (Figure 4A), and the diamond lying on the left axis.

### Postoperative Length of Stay (days)

The postoperative length of stay was used as an outcome indicator in seven of the included papers' statistical analyses of 599 patients with decreased left ventricular function. Levosimendan and IABP groups had 295 and 304 instances, respectively, out of the total. A fixed-effects model was utilized for the analysis since the Q-test for heterogeneity and the meta-analysis with MD as the effect size produced inconsistent results between effect sizes (*I^2^* = 97%), showing significant inter-study heterogeneity. The following are the findings of the meta-analysis: effect size MD = - 1.14, 95% CI: -1.33 to -0.95, *P* < 0.00001 (Figure 4B), with a diamond lying on the left axis, even while the postoperative length of stay (days) was shorter in the levosimendan group than that in the IABP group, the difference between the two groups was statistically significant (*P* < 0.00001).

### Ventilation Time (hours)

Utilizing breathing time as an outcome measure, statistical analysis was performed on 561 patients with reduced left ventricular function in seven of the included articles. Of these, the levosimendan and IABP groups each included 276 and 285 instances. The results of a meta-analysis using MD as the effect size and the Q-test for heterogeneity revealed inconsistent outcomes for different effect sizes (*I^2^* = 0%). The diamond fell on the left side of the axis with an effect size of MD = -0.72, 95% CI: -1.27 to -0.16, *P* = 0.01 (Figure 4C); this means that ventilation duration was shorter in the levosimendan group than in the IABP group. *P* < 0.05 indicates that there was no statistically significant difference between the two groups.

### Mean Arterial Pressure Levels

In five of the included publications, 432 individuals with compromised left ventricular function were statistically assessed using MAP levels as an outcome indicator. Of these, the levosimendan and IABP groups each included 240 and 192 instances, respectively. A fixed effects model was selected for the analysis since the meta-analysis using MD as the effect size and the Q-test for heterogeneity revealed diverse results between effect sizes (*I^2^* = 0%), showing reduced heterogeneity between trials. The meta-analysis' findings were as follows: the diamond fell on the left side of the axis, meaning that MAP levels were considerably lower in the levosimendan group than in the IABP group, and effect size of MD = -4.55, 95% CI: -6.14 to -2.96, *P* < 0.00001 (Figure 4D), having a difference between the two groups that is statistically significant (*P* < 0.05).

### Subgroup Analysis of Postoperative Intensive Care Unit Stay

Comparisons were made between the levosimendan group and the IABP group: in the NRCT subgroup, which contained three studies with a total sample size of 71 patients, the levosimendan group may be found to have a shorter ICU stay compared to the IABP group (MD = -0.46, 95% CI: -0.82 to -0.09, *P* = 0.01), but the reliability of the results is somewhat compromised by the high degree of heterogeneity (*I^2^* = 94%). In the randomized controlled trial (RCT) subgroup, there were 244 patients in the IABP group and 235 patients in the levosimendan group, the levosimendan group had a significantly shorter ICU stay than the IABP group (MD = -0.90, 95% CI: -1.06 to -0.74, *P* < 0.00001), with small differences between studies (*I^2^* = 35%) and high reliability of results. In the subgroup aged > 60 years, differences in ICU stay between the two groups were not significant (MD = 0.81, 95% CI: 0.02 to 1.59, *P* = 0.04), and the reliability of the results was low due to high heterogeneity (*I^2^* = 96%). In the subgroup aged < 60 years, there were 274 patients in the IABP group and 265 in the levosimendan group, with a heterogeneity of 22%. The levosimendan group had a significantly shorter ICU stay than the IABP group in this subgroup (MD = -0.89, 95% CI: -1.03 to -0.74, *P* < 0.00001).

**Fig. 5:**
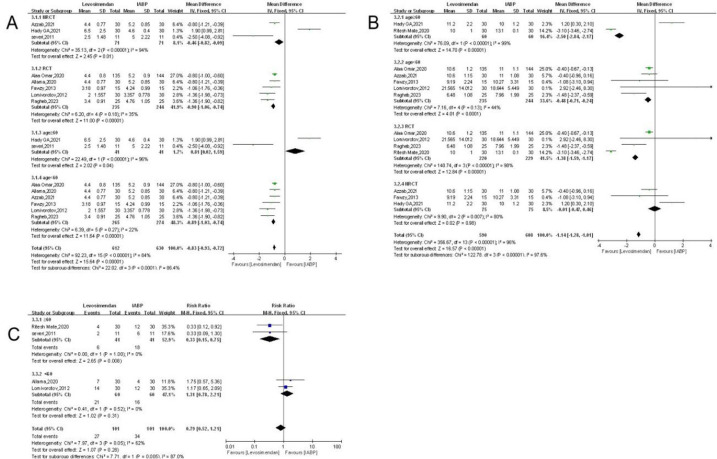
A) Forest plot of subgroup analysis of postoperative intensive care unit stay. B) Forest plot of subgroup analysis of postoperative length of stay. C) Forest plot of subgroup analysis of the incidence of atrial fibrillation. CI=confidence interval; IABP=intra-aortic balloon pump; IV=intravenous injection; NRCT=non-randomized controlled trial; RCT=randomized controlled trial; SD=standard deviation.

Overall, across all 1,242 patients (MD = -0.83, 95% CI: -0.93 to -0.72), with a highly significant overall effects test (*P* < 0.00001), there was significant heterogeneity among all studies (*I^2^* = 84%) and among subgroups (*I^2^* = 86.4%), as illustrated in Figure 5A.

### Subgroup Analysis of Postoperative Length of Stay

The postoperative length of stay was statistically analyzed in patients from different subgroups. Study designs varied, such as RCT and NRCT, and subgroups were also made according to age.

The levosimendan and IABP groups were compared. In the subgroup aged ≥ 60 years (total of 120 patients), the levosimendan group may have a shorter hospital stay compared to the IABP group (MD = -2.50, 95% CI: -2.84 to -2.17, *P* < 0.00001), but the reliability of the results is somewhat compromised by the high degree of heterogeneity (*I^2^* = 99%). In the subgroup aged < 60 years, there were 235 patients in the levosimendan group and 244 patients in the IABP group, with 44% heterogeneity and a significant overall effect (MD = -0.48, 95% CI: -0.71 to -0.24, *P* < 0.0001). In the RCT subgroup with 220 patients in the levosimendan group and 229 patients in the IABP group, the levosimendan group may have had a shorter postoperative hospital stay relative to the IABP group (MD = -1.38, 95% CI: -1.59 to -1.17, *P* < 0.00001), but there was a very high degree of heterogeneity (*I^2^* = 98%), with a highly significant test of overall effect. In the NRCT subgroup with a total of 85 patients, heterogeneity was 80%, and the overall effect test was not significant (MD = -0.01, 95% CI: -0.47 to 0.46, *P* = 0.98). The overall effects test was highly significant (*P* < 0.00001). There was significant heterogeneity between all studies (*I^2^* = 96%) and between subgroups (*I^2^* = 97.6%), as indicated in Figure 5B.

### Subgroup Analysis of Atrial Fibrillation

Subgroup analyses of AF were performed as follows: studies were divided into two subgroups according to age (≥ 60 years and < 60 years).

The levosimendan and IABP groups were compared. In the age ≥ 60 years subgroup, there were 41 patients with no heterogeneity (*I^2^* = 0%), the risk of AF may be lower with levosimendan group compared to IABP group (RR= 0.33, 95% CI: 0.15 to 0.75, *P* = 0.008), suggesting that in this subgroup, the levosimendan group may have an advantage in the prevalence of AF over the IABP group. In the subgroup aged < 60 years, there were 120 patients, with no heterogeneity (*I^2^* = 0%). Differences in risk of AF between levosimendan and IABP are not clear-cut and RR = 1.31, 95% CI: 0.78 to 2.21, *P* = 0.31. Overall, there was no significant difference between the levosimendan group and the IABP group in the risk of AF, there was some heterogeneity between studies (RR = 0.79, 95% CI: 0.52 to 1.21), and the overall effect test was not significant (*P* = 0.28). There was significant heterogeneity between all studies (*I^2^* = 62%) and between subgroups (*I^2^* = 87%). This is shown in Figure 5C.

### Publication Bias

**Fig. 6:**
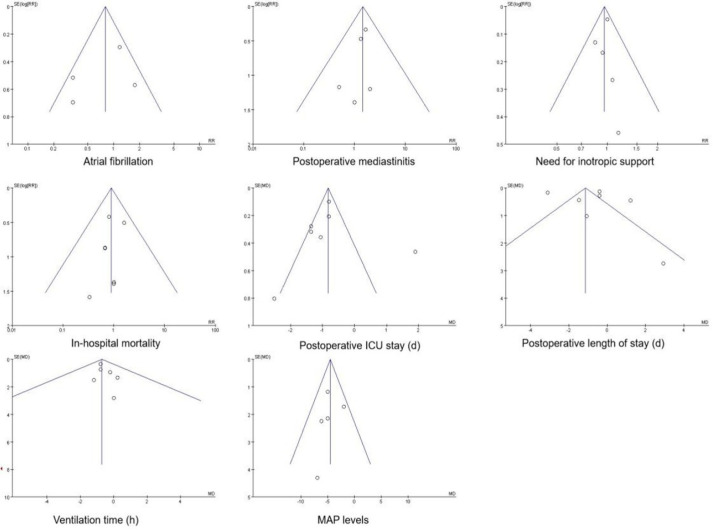
Funnel plots of publication bias. ICU=intensive care unit; MAP=mean arterial pressure; RR=relative risk; SE=standard error.

A funnel plot of the outcome markers for patients with decreased left ventricular function who were given levosimendan as opposed to IABP is shown in Figure 6. The findings demonstrated that this study was less likely to have publication bias when AF, postoperative mediastinitis, need for inotropic support, in-hospital mortality, postoperative ICU stay, ventilation time, and MAP levels were used as indicators. In contrast, when postoperative hospitalization time was used as an indicator, the scatter distribution of the study was more asymmetric on both sides of the funnel plot, and therefore publication bias may have existed.

### Sensitivity Analysis

**Fig. 7:**
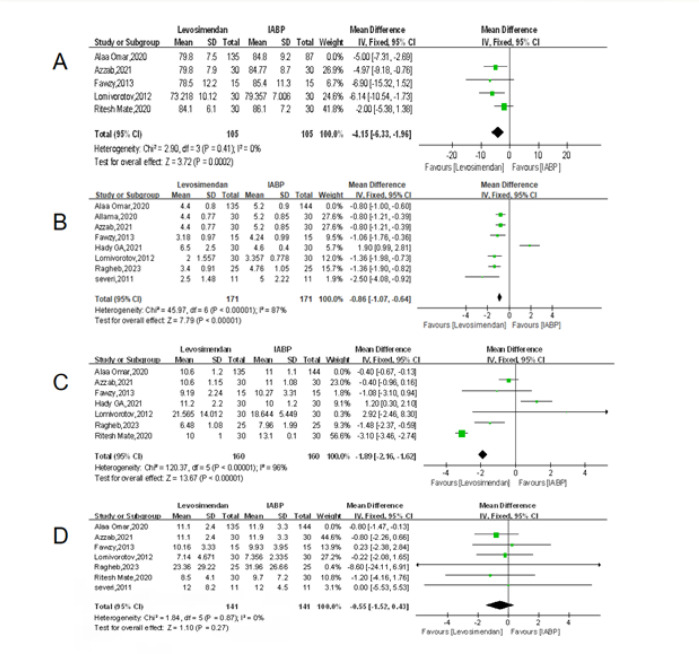
A) Forest plot of sensitivity analysis for mean arterial pressure levels. B) Forest plot of sensitivity analysis for postoperative intensive care unit stay. C) Forest plot of sensitivity analysis for postoperative length of stay. D) Forest plot of sensitivity analysis for ventilation time. CI=confidence interval; IABP=intra-aortic balloon pump; IV= intravenous injection; SD=standard deviation.

To evaluate the robustness of the main findings, sensitivity analyses were conducted by excluding the largest study, which contributed the greatest weight in several primary outcomes (over 50%). After exclusion, the benefit of levosimendan on postoperative ICU stay remained statistically significant (MD = -0.86; 95% CI: -1.07 to -0.64; *P* < 0.00001), consistent with the primary analysis, although heterogeneity persisted (*I^2^* = 87%). Detailed results of the sensitivity analysis for each outcome (including MAP levels, postoperative ICU stay, postoperative length of stay, and ventilation time) are presented in Figure 7. Similarly, for postoperative length of stay, the effect size remained stable (MD = -1.89; 95% CI: -2.16 to -1.62; *P* < 0.00001), again confirming the robustness of the findings despite high heterogeneity (*I^2^* = 96%). For MAP levels, the exclusion of the study by Omar et al.^[10]^ did not materially alter the outcome (MD = -4.15; 95% CI: -6.33 to -1.96; *P* = 0.0002), and heterogeneity was minimal (*I^2^* = 0%), indicating a consistent hemodynamic benefit. In contrast, the effect on ventilation time remained non-significant after exclusion (MD = -0.55; 95% CI: -1.52 to 0.43; *P* = 0.27), in line with the original analysis.

These analyses confirm that the observed effects of levosimendan on ICU stay, hospital stay, and MAP levels are stable and not unduly influenced by the largest included trial. The non-significant finding for ventilation time also remained unchanged, enhancing confidence in the consistency and validity of the pooled results.

### Intervention Characteristics

Considerable heterogeneity was observed among included studies regarding the administration protocols of levosimendan and IABP. Most trials initiated levosimendan infusion intraoperatively following anesthesia induction at a continuous dose of 0.1 µg/kg/min, with or without a loading dose of 12 µg/kg over 10 minutes. A minority of trials, such as the study by Ritesh Mate et al.^[11]^, employed preoperative administration 24 hours in advance. Similarly, IABP insertion timing ranged from 24 hours preoperatively to intraoperative initiation, and duration varied from 24 to 48 hours postoperatively or until hemodynamic stability was achieved. Detailed comparisons of administration timing, dosage, and duration across all included studies are summarized in Table 2. These discrepancies may confound pooled outcome interpretation. Standardization of administration protocols in future studies would be instrumental in minimizing inter-study variability and enhancing the comparability of results.

**Table 2:** Comparison of timing, dosage, and duration of levosimendan and IABP administration.

Study	Intervention	Timing	Dosage/Device	Duration
Severi ^[15]^	Levosimendan	Intraoperative	0.1 µg/kg/min (no loading dose)	24 h
	IABP	Preoperative	Fidelity 8F, 40 mL	≥ 48 h
Ritesh Mate ^[11]^	Levosimendan	24 h before operation	Total dose 200 µg/kg, 2 mL/h	24 h
	IABP	24 h before operation	Arrow 8F	Until hemodynamic stabilization
Lomivorotov^[9]^	Levosimendan	16 - 18 h before operation	12 µg/kg loading + 0.1 µg/kg/min	24 h
	IABP	16 - 18 h before operation	Arrow 8F, 40 mL	Until hemodynamic stabilization
Fawzy ^[17]^	Levosimendan	Intraoperative	12 µg/kg loading + 0.1 - 0.2 µg/kg/min	24 h
	IABP	Preoperative	Datascope 7.5F, 40 mL	≥ 24 h
Hady GA^[13]^	Levosimendan	Post-induction	0.1 µg/kg/min (no loading dose)	24 h
	IABP	At induction	Datascope 6.5 - 7.5F, 40 mL	Until hemodynamic stabilization
Azzab^[14]^	Levosimendan	Post-induction	Not specified; presumed 0.1 µg/kg/min	24 h
	IABP	At induction	N/A	Until hemodynamic stabilization
Allama ^[16]^	Levosimendan	Post-induction	To hemodynamic stabilization (dose not given)	To hemodynamic stabilization
	IABP	N/A	N/A	To hemodynamic stabilization
Ragheb^[12]^	Levosimendan	12 - 24 h before operation	12 µg/kg loading (10 min) + 0.1 µg/kg/min	24 h
	IABP	Intraoperative	Not specified (inserted as needed)	Until hemodynamic stabilization
Alaa Omar ^[10]^	Levosimendan	Post-induction	0.1 µg/kg/min (no loading dose)	24 h
	IABP	Post-induction	N/A	Until hemodynamic stabilization

IABP=intra-aortic balloon pump; N/A=not applicable

## DISCUSSION

CAD encompasses a spectrum of conditions, including stable and unstable angina, MI, and sudden cardiac death. These conditions arise from the narrowing or occlusion of blood vessels due to atherosclerotic plaques within the coronary arteries^[18]^. CAD can also lead to sudden mortality through various risk factors such as heart attack, myocardial injury, and arrhythmias^[19-21]^. CABG is currently considered the "gold standard" treatment for CAD^[22-26]^, and remains the primary therapeutic approach^[27,28]^. CABG aims to improve coronary perfusion and myocardial oxygenation by rerouting blood flow distal to any stenosis or obstruction^[29-31]^. Despite claims that CABG is cost-effective and associated with positive outcomes, particularly regarding short-term mortality^[32]^, complications such as acute renal failure, cardiac ischemia, arrhythmias, stroke, and others have been reported^[24,33-36]^.

Clinical factors such as life expectancy, presence of comorbid diabetes mellitus or chronic kidney disease, and degree of stenosis of the target vessel should be taken into account when selecting a CABG. Decades of clinical data confirm that the application of the left internal mammary artery (LIMA) as a bridging vessel for the left anterior descending artery (LAD) prolongs survival. These data come from observational studies, most of which were obtained before the introduction of optimal pharmacological treatment. Currently, LIMA is usually the first choice unless there are specific contraindications. If bridging vessels for the LAD are not available or bilateral internal mammary arteries (i.e., use of both the right internal mammary artery and LIMA) are selected, direct CABG can result in dramatic changes in mechanics and increased myocardial injury. Direct CABG can result in dramatic changes in mechanics and increased myocardial injury. In recent years, patients undergoing CABG have been characterized by advanced age, preoperative comorbidities, and impaired left ventricular function. These patients are referred to as "high-risk" patients because they have significantly reduced cardiac tolerance^[36]^.

Cardiothoracic surgeons face challenges with an increasing percentage of critically ill patients exhibiting poor function^[28]^, and determining the best method to utilize surgical and interventional resources to deliver the least invasive yet most effective long-term care remains a topic of intense debate. Although the levosimendan group showed statistical superiority in ICU length of stay, this metric is susceptible to multiple perioperative confounders and lacks uniformity, making it less reliable as a primary endpoint^[37]^. Therefore, the superiority of levosimendan over IABP based on differences in ICU length of stay alone is insufficient and needs to be judged in conjunction with harder endpoints (*e.g.*, mortality)^[38]^.

To reduce this risk, IABP, a form of internal counterpulsation, has been used as an adjunctive circulatory support device^[39]^. Acute left heart failure and low CO after CABG can be treated with IABP^[40]^, which provides circulatory support to hemodynamically unstable patient^[41]^. Prophylactic placement of the IABP has been shown to increase coronary perfusion and reduce myocardial oxygen consumption and left ventricular pressure overload through diastolic inflation and *in vivo* balloon contraction deflation, which may protect against critical cardiac perfusion^[42]^. IABP also promotes opening of collateral circulation in areas of cardiac ischemia and increases myocardial blood flow in areas of impaired coronary perfusion, thereby reducing and limiting the extent of acute MI^[43]^. IABP appears to be ineffective for conferring a survival benefit when cardiogenic shock is present, though. In the presence of poor end-organ perfusion and severe hypotension, IABP does not adequately maintain a failing heart^[44]^.

Levosimendan is a novel calcium channel blocker that was first approved by Swedish regulatory authorities in 2000 for the stabilization of hemodynamics in patients with acute decompensated chronic heart failure. Besides promoting vasodilation by activating adenosine triphosphate-dependent potassium channels in vascular smooth muscle cells, this agent enhances cardiac contractility through calcium sensitization^[45]^. Due to levosimendan's lack of effect on diastole, ventricular filling and coronary perfusion remain unaffected^[46]^. Intravenous administration of levosimendan has been shown to improve cardiac function in patients with left heart failure. Clinically, levosimendan is recommended for the treatment of heart failure^[47]^. Levosimendan demonstrated a dramatic increase in CO and improvements in several hemodynamic parameters for patients undergoing cardiac surgery with significantly compromised systolic function and symptoms of congestive heart failure^[48]^. While earlier research suggests that the routine use of levosimendan in all cardiac surgical settings cannot be recommended at this time^[49]^, certain patient subsets, such as those with poor ejection fraction or undergoing isolated CABG, may experience considerable mortality benefits^[50,51]^.

Levosimendan and IABP were compared in this meta-analysis for CABG in terms of efficacy and safety. Levosimendan outperformed IABP in the current meta-analysis among patients who underwent CABG. The univariate and multivariate analyses produced consistent results, indicating that levosimendan usage in CABG is a viable alternative. Levosimendan dramatically decreased MAP levels, postoperative length of stay, and postoperative ICU stays in patients, according to the study's findings. It also enhanced cardiac function by lowering afterload, contributing to better myocardial perfusion and oxygenation. Levosimendan usage prior to surgery may lessen the requirement for critical care and/or mechanical support^[52]^. Levosimendan appears to be the most beneficial medication for lowering postoperative mortality in cardiac surgery, according to one meta-analysis^[53]^. Another meta-analysis revealed that patients with decreased LVEF exhibited this benefit more prominently^[54]^. Also, postoperative mediastinitis occurred higher in the levosimendan group than in the IABP group, so its potential risks and benefits need to be carefully assessed when considering the use of levosimendan and further individualized treatment strategies may be required.

In addition, subgroup analyses revealed that levosimendan may be more effective than IABP in shortening postoperative ICU stays and length of postoperative hospital stay, especially in younger patients and in the RCT subgroup, which may contribute to improved postoperative recovery in these patients. This suggests that age may act as an effect modifier in the response to inotropic or mechanical support strategies. Younger patients may have preserved myocardial compliance and greater responsiveness to calcium-sensitizing agents like levosimendan^[55]^. Conversely, older individuals may have higher vascular stiffness, altered pharmacokinetics, or multiple comorbidities that attenuate the drug’s efficacy^[38,56]^. Further studies incorporating age-stratified analyses and pharmacodynamic assessments are warranted to elucidate these interactions. This finding suggests that the choice of adjuvant therapy in CABG may need to be tailored to the specific characteristics and needs of individual patients.

Sensitivity analyses were conducted to explore the influence of the largest included study on key outcomes. The results demonstrated that the favorable effects of levosimendan on postoperative ICU stay, hospital length of stay, and MAP levels remained statistically significant even after exclusion of this trial, suggesting that the overall findings were robust and not solely driven by a single study. This reinforces the reliability of the observed benefits of levosimendan in improving hemodynamic parameters and accelerating postoperative recovery.

However, the effect on ventilation time remained non-significant following exclusion, consistent with the primary analysis, indicating limited impact of levosimendan on this outcome. Despite the preservation of significance in several outcomes, heterogeneity remained substantial in ICU stay and hospital stay analyses, reflecting persistent variability in study protocols, patient populations, and intervention timing or dosing. These results underscore the importance of future trials employing standardized administration protocols and rigorous methodological quality to further clarify the clinical value of levosimendan in CABG patients^[57]^.

### Limitations

This meta-analysis has limitations, despite our meticulous adherence to the PRISMA recommendations. First, three studies were reported by the same authors at about the same time, and after careful reading, we eliminated two of the duplicates. Secondly, some included trials were of low quality and had relatively small sample sizes, which may have led to bias between our findings and the actual situation. Several included studies demonstrated unclear or high risk of bias, particularly in allocation concealment and blinding of participants and personnel. These methodological shortcomings, especially in non-randomized trials, may introduce systematic errors that could distort the true treatment effects. Moreover, this meta-analysis did not exclude low-quality studies, which may further affect the reliability of the pooled estimates. While inclusion of all available evidence allows for a more comprehensive synthesis, it also necessitates cautious interpretation of the results, especially when effect sizes are modest or derived from high-risk studies. Thirdly, some studies did not strictly follow the principles of randomization, control, and blinding, which affected the accuracy of the results. It is important to note that the large sample study by Omar et al.^[10]^, comprising 41.4% of MAPs and 53.1% of postoperative ICU stays, is the primary source of the findings of this meta-analysis. Finally, due to sample diversity, including differences in study populations as well as differences in surgical approaches, the results may be affected. For instance, the trial by Ritesh et al.^[13]^ required patients to have a LVEF of 25%, while other studies required an LVEF of 35%. Additionally, this study^[11]^ used off-pump CABG, whereas other studies used on-pump CABG.

## CONCLUSION

In conclusion, the use of levosimendan in CABG patients may reduce MAP levels and potentially shorten ICU stays compared to IABP. Additionally, levosimendan infusion could be advantageous for patients with severe aortic regurgitation or significant peripheral vascular disease, conditions which are absolute contraindications for IABP use^[10]^. However, the sample size of this meta-analysis was limited, and there is a scarcity of high-quality literature. The findings require further clinical evidence for robust support, and thus, caution is advised in clinical practice. Further investigation into the role of levosimendan in CABG patients is warranted in future studies. Therefore, further large-scale, high-quality RCTs with standardized intervention protocols are needed to confirm these findings and guide clinical decision-making.

## Data Availability

The authors declare that the data supporting the findings of this study are available within the article.

## Supplementary File 1

### Supplementary File 1

**Table:** Search Strategy

1. PubMed®: 1#(((simendan[MeSH Terms])) OR (levosimendan)) OR (intravenous levosimendan); 2#(intra-aortic balloon pump[MeSH Terms]) OR (Intra Aortic Balloon Pumping OR Intraaortic Balloon Pumping OR Balloon Pumping, Intraaortic OR Pumping, Intraaortic Balloon OR Pumping, Intra-Aortic Balloon OR Balloon Pumping, Intra-Aortic OR Pumping, Intra Aortic Balloon); 3#(coronary artery bypass[MeSH Terms]) OR (Artery Bypass, Coronary OR Artery Bypasses, Coronary OR Bypasses, Coronary Artery OR Coronary Artery Bypasses OR Coronary Artery Bypass Surgery OR Bypass, Coronary Artery OR Aortocoronary Bypass OR Aortocoronary Bypasses OR Bypass, Aortocoronary OR Bypasses, Aortocoronary OR Bypass Surgery, Coronary Artery OR Coronary Artery Bypass Grafting); 4#1 and#2 and #3
2. Embase: 1# 'levosimendan'/exp OR levosimendan OR (intravenous AND levosimendan) 2# 'intraaortic balloon pump'/exp OR 'intraaortic balloon pump' OR (balloon AND pumping, AND intraaortic) OR (pumping, AND intraaortic AND balloon) OR (pumping, AND 'intra aortic' AND balloon) OR (balloon AND pumping, AND 'intra aortic') OR (pumping, AND intra AND aortic AND balloon) 3# 'coronary artery bypass graft'/exp OR (artery AND bypass, AND coronary) OR (artery AND bypasses, AND coronary) OR (bypasses, AND coronary AND artery) OR (coronary AND artery AND bypasses) OR 'coronary artery bypass surgery' OR (bypass, AND coronary AND artery) OR (aortocoronary AND bypasses) OR (bypass, AND aortocoronary) OR (bypasses, AND aortocoronary) OR (bypass AND surgery, AND coronary AND artery) OR 'coronary artery bypass graft' 4# #1 AND #2 AND #3
3. Cochrane Database of Clinical Trials: #1 MeSH descriptor: [Simendan] explode all trees #2 ("levosimendan” or “intravenous levosimendan” or “intravenous levosimendan"):ti,ab,kw #3 #1 or #2 #4 MeSH descriptor: [intraaortic balloon pump] explode all trees #5 ("intra-aortic balloon pump or Intra Aortic Balloon Pumping or Intraaortic Balloon Pumping or Balloon Pumping, Intraaortic or Pumping, Intraaortic Balloon or Pumping, Intra-Aortic Balloon or Balloon Pumping, Intra-Aortic or Pumping, Intra Aortic Balloon"): ti,ab,kw #6 #4 or #5 #7 MeSH descriptor: [coronary artery bypass graft] explode all trees #8 ("coronary artery bypass graft or Artery Bypass, Coronary or Artery Bypasses, Coronary or Bypasses, Coronary Artery or Coronary Artery Bypasses or Coronary Artery Bypass Surgery or Bypass, Coronary Artery or Aortocoronary Bypass or Aortocoronary Bypasses or Bypass, Aortocoronary or Bypass, Aorto coronary or Bypass, Aortocoronary or Bypass Surgery, Coronary Artery or Coronary Artery Bypass Grafting"):ti,ab,kw #9 #7 or #8 #10 #3 and #6 and #9
4. Google Scholar: (levosimendan OR intravenous levosimendan) AND (intra-aortic balloon pump OR Intra Aortic Balloon Pumping OR Intraaortic Balloon Pumping OR Ballon Pumping, Intraaortic OR Pumping, Intraaortic Balloon OR Pumping, Intra-Aortic Ballon OR Balloon Pumping, Intra-Aortic OR Pumping, Intra Aortic Balloon) AND (coronary artery bypass OR Artery Bypass, Coronary OR Artery Bypasses, Coronary OR Bypasses, Coronary Artery OR Coronary Artery Bypasses OR Coronary Artery Bypass Surgery OR Bypass, Coronary Artery OR Aortocoronary Bypass OR Aortocoronary Bypasses OR Bypass, Aortocoronary OR Bypasses, Aortocoronary OR Bypass Surgery, Coronary Artery OR Coronary Artery Bypass Grafting)

## Artificial Intelligence Usage

The authors declare use of ChatGPT 4.0 for language polishing and stylistic optimization. The content produced by the artificial intelligence tool was revised and edited by the authors as necessary, and they take full responsibility for the content to be published.
